# Diagnosis and Surgical Treatment of Acute Pancreatitis

**Published:** 1909-12

**Authors:** Emory Lanphear

**Affiliations:** St. Louis, Mo. Professor of Surgery in the Hippocratean College of Medicine; Consulting Gynecologist to the Ellen Osborn Hospital


### TEXAS MEDICAL JOURNAL.

Established July, 1885

F. E. DANIEL, M. D., - - - - - - Editor, Publisher and Proprietor

Published Monthly.—Subscription $1.00 a Year.

Vol. XXV. AUSTIN, DECEMBER, 1909. No. 6

The publisher is not responsible for the views of the contributors.

### Original Articles.

For Texas Medical Journal.

### Diagnosis and Surgical Treatment of Acute Pancreatitis.

BY EMORY LANPHEAR, M. D., PH. D., LL. D., ST. LOUIS, MO.

Professor of Surgery in the Hippocratean College of Medicine; Consulting Gynecologist to the Ellen Osborn Hospital.

It seems that we have been rather slow in recognizing the importance of acute pancreatitis and realizing that it may be remedied by prompt operative treatment.

It is true that diagnosis is difficult—the symptoms being those of perforation-peritonitis or of acute ileus. The glycosuria accompanying chronic inflammation of the pancreas is absent, as are also the transverse tumor of the upper abdomen and the pronounced tenderness of the upper lumbar region.

Generally the first symptom is a slight pain in the belly just below the ensiform cartilage—not the sharp pain of an impacted gall-stone, but a dull aching with sickening sensation.

Soon after the onset of pain grave symptoms of intra-peritoneal trouble appear: rigidity of the upper part of the abdomen, frequently with swelling, vomiting, quick pulse, and a temperature of 100-102, with marked collapse.

The pain sometimes is intense, being most pronounced in the median line above the umbilicus, in which cases the prostration is likely to be extreme, the pulse thready and intermittent and the temperature above the average, running to 102 or 103. (In one of my worst cases, however, the temperature was subnormal.) These pains radiate to the back, and are associated with more tympanites than those with less epigastric distress.

Jaundice may appear, even though operation shows gall-stones to be absent—pressure on the ductus communis being the cause.

As in gall-stone colic, the vomiting may be severe, ejection of food being followed by bile, and rarely by small quantities of blood. Straining increases the discomfort at the epigastrium and may increase the distention. In rare instances emesis may be absent.

Hiccough is occasionally severe and distressing. It generally means death.

The presence of an obstinate constipation of several days' duration—for which the patient has taken physic—tends to lead the doctor to a diagnosis of obstruction of the bowels; though usually the obstruction is not absolute, gas being passed, and an enema may secure an evacuation. Later on a diarrhoea may supervene.

The expression of the patient's face is that of peritonitis—especially late in the disease.

Unlike the usual case of peritonitis, delirium is present for some hours before the end.

Acute pancreatitis may be suspected when a patient who has had no symptom of gall-stones or of gastric or duodenal ulcer is suddenly seized with severe pain at the epigastrium, rapidly spreading over the entire abdomen, followed by vomiting and collapse. The presence of a tumor (nearly always to be felt within twenty-four hours after the onset) below the ensiform aids in the diagnosis.

The differentiation is to be made from (a) intestinal obstruction, (b) perforation-peritonitis (gastric or duodenal), (c) ruptured gall-bladder or ducts, (d) abscess of gall-bladder, and (e) gangrenous appendicitis. Generally the diagnosis can be made by exclusion, but too long time should not be spent in this, as all these conditions are operative ones and delay means danger.

A peculiar feature of the disease is the extremely rapid loss of flesh.

Another odd symptom is a cyanosis of the abdominal wall, but it is not constant.

The infective agent usually is the bacillus coli communis—hence the low grade of fever, the not serious inflammation of peritoneum and the favorable prognosis if early drainage be secured. But in the fatal (fulminating) cases the streptococcus has been found.

As to treatment: Instant operation gives the only cure; but the gravity of the case must be explained to the friends, since people now expect so much from operation on the abdominal organs. The prognosis is very grave at best—only few patients recovering.

It is best to give a hypodermic injection of morphine (1-4 gr.) and hyoscine (1-100 gr.) at the onset, as this relieves the pain and checks the nausea as well as helping overcome the collapse. If time permit before operation, a second dose may well be given in an hour and a half after the first. Then only a trifling amount of chloroform or ether will have to be given—a matter of much importance in this disease where collapse is already so prominent.

In most instances, perhaps, there has been some hemorrhage from the surface of the pancreas (acute hemorrhagic pancreatitis), so that when the abdomen is opened there is found a large quantity of bloody serum and an enlarged pancreas with exudate about it. Examination of the pancreas shows an infiltration with numerous infarcts and some foci of fat-necrosis.

In the treatment the peritoneal cavity should be first irrigated and sponged out. Gauze packing should be carefully placed all around the infected organ, so that if incision has to be made later into the substance of the pancreas for liberation of pus, there shall be no contamination of peritoneum with pancreatic secretion—it having a powerfully destructive action on the serosa.

It is best to leave the incision wide open so as to afford perfect exit for all infected fluid. In favorable cases the general abdominal cavity will be shut off by adhesions within forty-eight hours, when incisions may be made into the substance of the pancreas if deemed necessary.

Robson advocates making a posterior incision (a free vertical one in the left costo-vertebral angle) for drainage whenever gangrene or large quantities of pus are found.

During the operation the gall-bladder and ducts should be carefully inspected and any stones or pus therein contained removed, for in many cases the trouble begins with an infection of the gall-tract. Sometimes, if the patient is strong enough to stand it, cholecystectomy should be performed, especially in abscess of the gall-bladder; but unfortunately the general condition is usually such that only incision, irrigation and insertion of drainage can be done.

On the other hand the experienced surgeon in operating for cholelithiasis will invariably examine the pancreas, regardless of presence or absence of symptoms pointing to pancreatitis.

In exploratory abdominal section for obscure, acute disease of the upper abdomen, the pancreas must always be examined—by dividing the ligamentum gastrocolicum. Generally it is best to see as well as feel the organ.

When suppurative pancreatitis is found the question of the advisability of immediate opening arises. On account of the infection of the peritoneum it is best to leave the abscess unopened if the condition of the patient will permit until protective adhesions can form. Generally the sepsis is so profound that evacuation of the abscess at time of operation is imperative; in which case the posterior opening should be made as advised by Moynihan for downward drainage, provided the abscess lies in such position that this method is practical. If not, the pus should be removed by sponging as it oozes through a small opening which later is enlarged to embrace the whole width of the cavity. It is best not to curet, or even mop too severely, the surface of the abscess-cavity as the infection would thereby be spread and some leakage of pancreatic fluid occur, each of which is very undesirable. A tube surrounded by unsoiled gauze is then to be introduced to the depths of the cavity and carefully tamponned around to prevent if possible leakage into the peritoneal cavity. But all of my cases thus treated have died.

In the hemorrhagic cases there is great danger of bleeding on removal of the gauze packs. Hence they should not be removed until very late in convalescence, the end of the second or even well into the third week.

Following operation there is usually an intense degree of collapse—demanding subcutaneous injection of normal saline solution, strychnine, glonoin, and even camphorated oil.

As soon as possible after operation free catharsis should be induced to rid the system of the toxic material already accumulated, for it is the toxemia rather than the local infection which causes a fatal termination.

---

Christmas Greeting.—The "Red Back" sends Christmas greeting to its hosts of friends and wishes them health, happiness and prosperity with the capital H's and a capital P.

---

Dr. A. R. Bowman, of Uvalde, was elected President of the Fifth Councilor District Medical Society at the recent meeting of that Society held in San Antonio, jointly with the Medical Society of the Southwest. Good.

